# Mode of delivery of Cognitive Behavioral Therapy for Insomnia: a randomized controlled non-inferiority trial of digital and face-to-face therapy

**DOI:** 10.1093/sleep/zsab185

**Published:** 2021-08-06

**Authors:** Håvard Kallestad, Jan Scott, Øystein Vedaa, Stian Lydersen, Daniel Vethe, Gunnar Morken, Tore Charles Stiles, Børge Sivertsen, Knut Langsrud

**Affiliations:** 1 Division of Mental Health Care, St. Olavs University Hospital, Trondheim, Norway; 2 Department of Mental Health, Norwegian University of Science and Technology, Trondheim, Norway; 3 Institute of Neuroscience, Newcastle University, Newcastle, United Kingdom; 4 Department of Health Promotion, Norwegian Institute of Public Health, Bergen, Norway; 5 Department of Psychology, Norwegian University of Science and Technology, Trondheim, Norway; 6 Department of Research and Innovation, Helse-Fonna HF , Haugesund, Norway

**Keywords:** Cognitive Behavioral Therapy for Insomnia, digital, face-to-face, non-inferiority, randomized controlled trial, insomnia disorder

## Abstract

**Study Objectives:**

Digital Cognitive Behavioral Therapy for Insomnia (dCBT-I) has demonstrated efficacy in reducing insomnia severity in self-referred and community samples. It is unknown, however, how dCBT-I compares to individual face-to-face (FtF) CBT-I for individuals referred to clinical secondary services. We undertook a randomized controlled trial to test whether fully automated dCBT-I is non-inferior to individual FtF CBT-I in reducing insomnia severity.

**Methods:**

Eligible participants were adult patients with a diagnosis of insomnia disorder recruited from a sleep clinic provided via public mental health services in Norway. The Insomnia Severity Index (ISI) was the primary outcome measure. The non-inferiority margin was defined a priori as 2.0 points on the ISI at week 33.

**Results:**

Individuals were randomized to FtF CBT-I (*n* = 52) or dCBT-I (*n* = 49); mean baseline ISI scores were 18.4 (SD 3.7) and 19.4 (SD 4.1), respectively. At week 33, the mean scores were 8.9 (SD 6.0) and 12.3 (SD 6.9), respectively. There was a significant time effect for both interventions (*p* < 0.001); and the mean difference in ISI at week 33 was −2.8 (95% CI: −4.8 to −0.8; *p* = 0.007, Cohen’s *d* = 0.7), and −4.6 at week 9 (95% CI −6.6 to −2.7; *p* < 0.001), Cohen’s *d* = 1.2.

**Conclusions:**

At the primary endpoint at week 33, the 95% CI of the estimated treatment difference included the non-inferiority margin and was wholly to the left of zero. Thus, this result is inconclusive regarding the possible inferiority or non-inferiority of dCBT-I over FtF CBT-I, but dCBT-I performed significantly worse than FtF CBT-I. At week 9, dCBT-I was inferior to FtF CBT-I as the 95% CI was fully outside the non-inferiority margin. These findings highlight the need for more clinical research to clarify the optimal application, dissemination, and implementation of dCBT-I.

**Clinicaltrials.gov:** NCT02044263: Cognitive Behavioral Therapy for Insomnia Delivered by a Therapist or on the Internet: a Randomized Controlled Non-inferiority Trial.

**Statement of Significance**

Direct comparisons between the current gold-standard and new digital therapeutics in clinical populations are needed to benchmark the effectiveness of digital therapeutics. This is the first study to compare a fully automated digital Cognitive Behavioral Therapy for Insomnia (CBT-I) with individual Face-to-Face (FtF) CBT-I in a clinical population. In this context, we could not conclude about long-term non-inferiority, although dCBT-I performed significantly worse than FtF CBT-I in reducing insomnia severity. Further research on dCBT-I may be useful to understand how best to deliver this treatment and who might best be served by it. Future developments may include tailoring the dCBT-I intervention to different populations.

## Introduction

Insomnia is a significant health problem, affecting 10%–15% of the general population [1]. Its diagnosis is primarily dependent on the presence of three phenomena: persistent sleep difficulty, adequate sleep opportunity, and associated daytime dysfunction for more than 3 months [2]. There is a consensus that Cognitive Behavioral Therapy for Insomnia (CBT-I) is the best intervention for insomnia and should be a first-line treatment option [3]. However, there is a significant gap between supply and demand which is largely attributable to the lack of availability of therapists trained in CBT-I [4]. To overcome barriers regarding access to face-to-face (FtF) therapy, CBT-I has been adapted for delivery via digital means such as websites or apps. These digital approaches (which we will refer to as dCBT) differ in the amount of support from clinicians that is offered, ranging from materials included only as a supplement to a course of FtF therapy, via therapist-guided programs, to fully automated dCBT [5].

Although dCBT-I models vary, a recent meta-analysis of 11 randomized controlled trials (RCTs) indicated statistically significant effects on insomnia severity (pooled effect size (ES) > 1.0) for dCBT-I compared with control interventions such as sleep hygiene [6]. Further, in a recent large-scale RCT of fully automated dCBT-I in the general population, we observed an ES (Cohen’s *d*) of 1.2 for insomnia severity, which is similar to that reported in trials of FtF CBT-I [7]. These data offer robust support for the wider community access to fully automated dCBT-I [8]. However, a critical issue still needs to be addressed: While the development of dCBT-I was never intended to replace FtF treatment in clinical settings, the issue of therapist availability has increased calls for investigators to establish how dCBT-I performs relative to gold-standard FtF therapies [9] in patients with a diagnosis of insomnia disorder rather than focusing solely on convenience samples of individuals with symptoms of insomnia recruited from the community [6].

Cognitive behavioral therapies are established interventions for most common mental disorders, which has encouraged the development and analysis of different modalities for delivery. One recent meta-analysis of guided and unguided digital, and FtF delivery of CBT for depression found that digital CBT was superior to FtF CBT in improving depressive symptoms [10]; furthermore, other reviews of therapist-guided digital CBT versus FtF CBT for psychiatric or somatic disorders found no differences in outcomes between the two modalities [11, 12]. Overall, this suggests that digital CBT may have similar effects as FtF CBT for individuals with various psychiatric and somatic disorders. However, for individuals with insomnia, there are only three published RCTs comparing dCBT-I with FtF CBT-I [13–15]. Taken together, the RCT findings are inconclusive regarding the effects on insomnia severity, as one found a trend favoring individual FtF CBT-I but no significant differences between the two modalities [15], one trial indicated that guided dCBT-I achieved similar improvements to group FtF CBT-I [13], and one reported that individual FtF CBT-I was superior to guided dCBT-I [14]. These trials recruited convenience samples via media [13, 14] or, for example, subpopulations of active military personnel [15], which means that the findings may not be generalizable to secondary care patients. Another two trials used guided rather than a fully automated dCBT-I. In some ways, a guided version of dCBT-I can be regarded as a hybrid between FtF and a fully automated dCBT-I, as guided dCBT-I provides additional direct therapeutic input and support to the individual (rather than being practiced independently).

The current study focuses on the critical gaps in the evidence-base regarding dCBT-I, namely, how does a fully automated dCBT-I intervention compare with individual FtF CBT-I when applied to a clinical secondary care population? To address this issue, we used an established, efficacious dCBT-I program called Sleep Healthy Using The internet (SHUTi) [16] and compared it with individual FtF CBT-I delivered by experienced therapists. Further, we recruited adult patients with sleep problems that met diagnostic criteria for insomnia disorder who were referred to a sleep clinic because these difficulties were impairing their functioning and/or quality of life.

Given the lack of comparative RCTs of gold standard individual FtF CBT-I (reference therapy) and fully automated dCBT-I (new therapy), we opted to conduct a non-inferiority trial with two arms. This design is warranted when a new treatment has advantages such as greater availability or reduced cost than an established gold standard (reference) [17], as the premise is that a non-inferiority RCT can determine that the new intervention is no worse than the reference intervention by a predefined “acceptable amount” [17].

In sum, the primary aim of this RCT was to test if dCBT-I is non-inferior to individual FtF CBT-I on insomnia severity as measured at six-months follow-up. The six-month follow-up was chosen as the primary endpoint for the trial because longer-term outcomes were regarded to be of higher relevance for both patients and health care systems. Secondary aims (defined a priori), involved superiority analyses to (1) estimate rates of clinical response and remission of insomnia according to the group, and (2) explore if there were any between-group differences in psychological distress, fatigue, or self-reported sleep-wake patterns.

## Methods

### Study design

A parallel-group randomized controlled non-inferiority trial of dCBT-I versus FtF CBT-I, with participants assigned in a 1:1 ratio. The protocol was approved by the Regional Ethical Committee of South-East Norway (Reference: 2013/1836) and the RCT was registered on the Clinical Trials website (ClinicalTrials.gov: NCT02044263).

**Figure 1.**
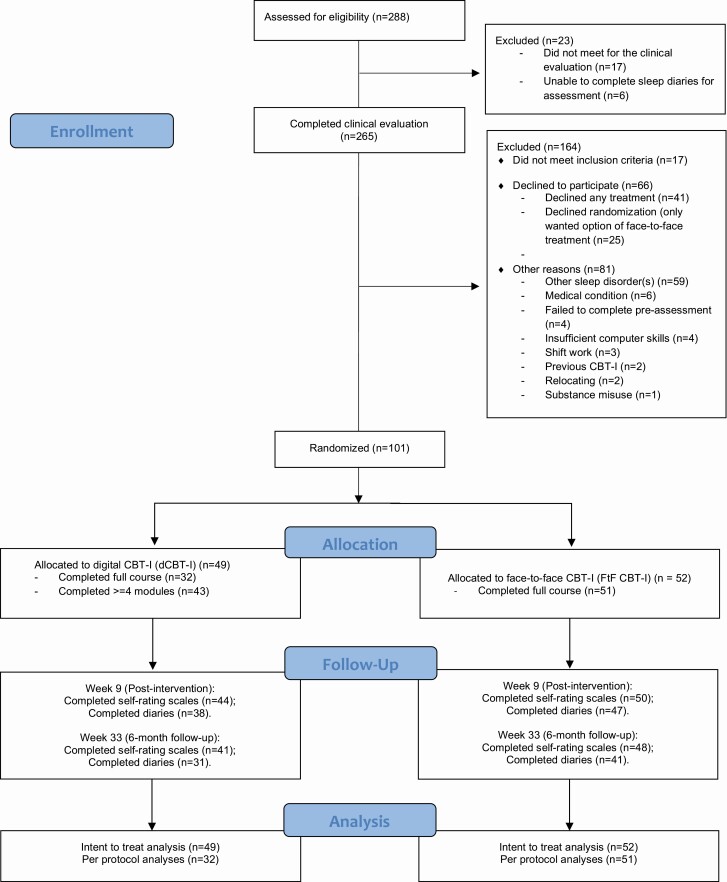
Flow diagram of trial: participant inclusion, timing of assessments, and completion rates.

The trial follows the CONSORT guidelines for a non-inferiority trial [17]. The flowchart is shown in Figure 1 and the CONSORT checklist is provided in the online Supplementary Materials (see Appendix 1).

### Participants

All RCT participants provided written informed consent. Between October 2014 and January 2016, patients referred for treatment of insomnia to a secondary care sleep clinic at St. Olavs University Hospital, Trondheim, Norway were offered the opportunity to be involved in the RCT. Participants who completed all assessments were offered a gift voucher worth NOK 500 (equivalent to EUR 50) as a reimbursement for their time, etc.

Eligibility criteria: Eligible participants were all patients who had been referred to the sleep clinic with a presentation of insomnia. Participants could be included if they were aged >= 18, the presentation met diagnostic criteria for insomnia disorder as described in the DSM-5 [2], they had regular internet access and self-reported proficiency in basic computer/internet skills (as required to participate in the RCT and complete online assessments, etc.), and they were willing and able to provide written informed consent.

The diagnosis of insomnia disorder was assessed by (1) a trained psychiatrist or licensed clinical psychologist using a semi-structured interview based on the Insomnia Interview Schedule [18], supplemented by a module assessing circadian rhythm disorders, and (2) completion of a pre-assessment sleep diary that recorded sleep-wake cycle patterns for the 14 days prior to the interview. The interview also assessed current sleep-wake pattern, sleep history, previous history and treatment of sleep problems (including medication), functional sleep analysis (e.g. daytime consequences of sleep problems and perpetuating factors), and history of somatic and psychiatric illness and treatment.

Individuals were excluded if they met one or more of the following criteria: evidence of circadian rhythm disorder, or an organic sleep disorder as assessed in the Insomnia Interview Schedule; or, for sleep apnea specifically (established via evidence of sleep apnea at interview assessment or an Oxygen Desaturation Index above a cutoff of 9 as assessed by oximetry recordings) [19]; and/or a current alcohol and/or substance misuse problem. Also excluded were individuals: working night shifts and unable to discontinue this work pattern during the RCT, with previous exposure to CBT-I, or with a medical condition where sleep restriction is deemed inappropriate due to a potential for worsening of the medical condition (e.g. an attack phase of multiple sclerosis or epilepsy), and/or with insufficient fluency in Norwegian (i.e. unlikely to be able to complete interventions).

### Randomization

The randomization program was designed and implemented by the Section of Applied Clinical Research, at the Faculty of Medicine and Health, the Norwegian University of Science and Technology (NTNU). The randomization utilized a balanced allocation sequence using blocks of randomly varying size. After trial completion, it was revealed that the range of blocks was between 6 and 20. Specifically, the first block was 20 (10 + 10), and the subsequent blocks varied between 6 (3 + 3) and 8 (4 + 4). Participants were randomized by the three clinicians who undertook the screening assessment (and who delivered FtF CBT-I). The clinicians logged on to an online portal and eligible participants were then assigned to one of the two groups. The clinicians could not influence the process in any way and the result of the randomization was communicated directly to the patient via email.

Individuals randomized to FtF CBT-I received an outpatient appointment for the sleep clinic. Participants randomized to dCBT-I were provided with a link to allow them to login to the online program. As this intervention was fully automated and delivered online, there was no contact between participants and the professional at the sleep clinic. If problems arose with the delivery of the online program or the completion of assessments, a technician could offer support to the participant.

### Procedures

#### Individual face-to-face CBT-I.

CBT-I is a multicomponent treatment and consisted of the following interventions: psychoeducation about sleep and sleep hygiene, sleep restriction, stimulus control, and challenging beliefs and perception of sleep [18, 20]. Table 1 provides a description of the week-by-week interventions. The course of FtF CBT-I involved three to eight sessions delivered over 6–9 weeks [18, 20], although the exact number of sessions provided is dictated by client progress. Three therapists provided FtF CBT-I (two authors [H.K. and K.L.]; and another a licensed psychologist); all had participated in training courses, had 3–10 years post-graduate experience in CBT-I and received ongoing supervision in CBT-I. Their adherence to the therapy protocol during the RCT was monitored via weekly review meetings.

**Table 1.** An overview of the content of each core of dCBT-I and the content of sessions in the FtF CBT-I

Core/session	dCBT-I	FtF CBT-I
1	Overview: Reviews the nature of insomnia and how the program works; participants identify their sleep problems and set up personal treatment goals.	Motivation and personal treatment goals. Psychoeducation about sleep architecture and the two-process theory of sleep-wake regulation. Education about sleep hygiene if patient is engaging in activities that could obviously interfere with the effect of sleep restriction (e.g. excessive caffeine use). Setting up sleep restriction (lower limit of 5 h). Setting up a plan for tapering of sleep medication if a treatment goal for the patient is to stop or reduce medication use.
2	Behavior and sleep: Focuses on how behavioral changes can improve sleep, with special emphasis on sleep restriction (lower limit of 5 h).	Review of adherence to sleep restriction and problem solving if needed. Socratic dialogue about changes in beliefs and behaviors about sleep, particular changes that have occurred as a function of sleep restriction (e.g. the need for safety behaviors in order to sleep). Motivational work to keep the patient adhering to sleep restriction.
3	Behavior and sleep 2: Focuses on behavioral changes that can improve sleep, with special emphasis on stimulus control	As week 2. Adding stimulus control if necessary.
4	Sleep and thoughts: Focuses on addressing and changing beliefs and thoughts that might impair sleep.	As weeks 2 and 3.
5	Sleep hygiene: Teaches about lifestyle and environmental factors that might interfere with sleep (e.g. caffeine and nicotine intake, electronic media use in bed).	As weeks 2 and 3.
6	Relapse prevention: Focuses on integrating the behavioral, educational, and cognitive components from the previous cores to develop strategies to prevent future episodes of poor sleep to develop into full-blown chronic insomnia.	Final session. Evaluation of current status relative to treatment goals in session 1. Relapse prevention: Check that the patient has understood the rationale behind sleep restriction and can implement use of sleep diaries and sleep restriction should sleep problems occur later. Implement stimulus control if the patient wants to stop sleep restriction.

#### Digital CBT-I.

We employed a dCBT-I program entitled SHUTi [21] that was created by investigators at the University of Virginia and was translated into Norwegian by the Norwegian Institute of Public Health. The program incorporates the same approaches used in standard (FtF) CBT-I packages, but the educational, behavioral, and cognitive interventions are conceptualized as six “cores” [22]. Participants with a goal of reducing or eliminating sleep medication use were informed that they should discuss this with the prescribing physician. Each core is accessed in a predefined sequence and admittance to subsequent cores is based on time (1 week after the completion of the previous core), and a required five diaries must be entered in the previous 7 days to move from core 1 to core 2 to set the program sleep window. Each core typically takes 45 to 60 min to complete, but there were no specific instructions about how long time the participants should take, and again completion may be partly affected by comprehension, attention, etc. Participants had access to the intervention for 6 months.

### Assessments

Prior to randomization, background information was collected regarding demography (age, sex, socioeconomic, and employment status), while comorbid mental disorders were assessed with the self-reported Psychiatric Diagnostic Screening Questionnaire Comorbid Mental Disorders (PDSQ) [23]. The PDSQ consists of 111 items assessing symptoms of common *DSM-IV* Axis I disorders encountered in outpatient mental health settings (see Supplementary Table S1). Comorbid physical disorders, past and/or current mental health treatment, and past and/or current use of sleep medications, and number of different agents prescribed were identified via the clinical interview augmented by medical casenote recordings.

At baseline, 9 weeks after randomization (post-intervention), and 33 weeks after randomization (6 months post-intervention), participants were asked to complete self-report assessments. Insomnia severity was assessed using the Insomnia Severity Index (ISI) [24]. Scores on this 7-item questionnaire range from 0 to 28 with higher scores indicating greater symptom severity. The ISI has good psychometric properties, is widely used, and is a recommended primary outcome measure in insomnia research [25]. A reduction in ISI scores of 8 or more points from baseline is regarded as a response to treatment, and an absolute score on the ISI of 7 points or less is regarded as remission. Data on daily sleep-wake parameters were collected using an online version of the consensus sleep diary [26]. Individuals were asked to record information about sleep onset latency (SOL), wake after sleep onset (WASO), early morning awakenings (EMA), number of nocturnal awakenings, total sleep time, and sleep efficiency for 10 days (out of the previous 14 consecutive days). Psychological distress was assessed using the 14-item version of the Hospital Anxiety and Depression Scale (HADS). The HADS has been shown to reliably rate symptoms of psychological distress in hospital outpatient clinics [27]. Scores range from 0 to 42 with higher scores indicative of greater distress. Daytime fatigue was assessed using the 13-item Chalder Fatigue Scale (CFS). Scores range from 0 to 39, with higher scores indicative of greater psychological and physical fatigue [28]. Dysfunctional beliefs about sleep were assessed using the Dysfunctional Beliefs and Attitudes about Sleep scale—16 items (DBAS-16) [29]. Mean scores on these items range from 1 to 10 with higher scores indicating a higher endorsement of dysfunctional beliefs about sleep.

### Statistical analyses

All primary and secondary outcomes reported here are based on Intent-To-Treat (ITT) analyses. The primary outcome was the ISI, and the primary endpoint was at week 33. The non-inferiority margin for the mean difference in ISI scores between the two interventions was defined a priori as <2-points.

With an assumed standard deviation (SD) of 4.0, a difference of 2 points on the ISI corresponds to a moderate ES (Cohen’s *d* = 0.5). Given the usual magnitude of ES in RCTs of interventions for insomnia (>1.0) and the previously employed margins for non-inferiority (e.g. 4 points on the ISI), this margin is likely to be enough to separate a change in mean ISI scores that is clinically important from a change that has limited clinical relevance [6, 13, 14, 30]. Under these assumptions, we estimated that a sample size of 100 participants commencing the RCT would give a power of 80% for non-inferiority of dCBT-I (α = 0.05) in the ITT analyses.

We interpreted the primary outcome from the 95% Confidence Interval (CI) of the estimated between-group differences on the ISI as recommended in the CONSORT guidelines for non-inferiority trials [17]. That is, if the 95% CI for the mean difference between the two intervention groups was between −2 and ∞, then we would declare that dCBT-I is *non-inferior* to individual FtF CBT-I. If the 95% CI was between −∞ and 0, then we would declare that individual FtF CBT-I is *superior* to dCBT-I. If the 95% CI was between 0 and ∞, then we would declare that dCBT-I is *superior* to individual FtF CBT-I. The secondary outcome of between-group differences on the ISI at week 9 was also interpreted using the same non-inferiority margin. Other secondary outcomes included between-group differences in HADS, CFS, and sleep variables recorded in the diary on the assessment at week 9 and week 33, and differences in numbers of remitters and responders based on ISI scores at weeks 9 and 33. These secondary outcomes were interpreted with standard superiority guidelines as suggested in the CONSORT non-inferiority guidelines [17].

We used SPSS version 25 for analysis of the primary and secondary outcomes. These analyses were performed by a statistician (SL) who was blinded to group allocation. We used a linear mixed model with individual as the random effect, time and group and their interaction as categorical covariates, and ISI score as the dependent variable. This approach implicitly accounts for missing-at-random data. We adjusted for the baseline value of the outcome variable, and estimated the difference in ISI scores between groups at week 9 and week 33 from the interaction terms, as recommended by Twisk et al. [31].

Superiority analyses of group differences in CFS, HADS and DBAS mean scores, and self-reported sleep-wake cycle patterns (i.e. sleep diary parameters) were performed using similar linear mixed models as above. Further, the proportions of participants per group who met ISI criteria for response and remission were compared using Pearson’s chi-squared test and the Newcombe Hybrid Score CI [32].

The above approaches for the continuous variables were repeated for the per protocol (PP) analyses restricted to patients who completed all therapy sessions they were offered and all modules of dCBT-I as recommended in the CONSORT non-inferiority guidelines [17].

Cohen’s *d* was estimated as the difference in mean scores divided by the baseline SD [33]. For the between-group effect sizes, this was calculated using the difference estimate from the mixed model analyses divided by the pooled SD at baseline. For the within-group effect sizes, this was calculated using the difference in mean scores at baseline and each follow-up assessment divided by the within-group SD at baseline for each condition.

## Results

Of 288 potential participants, 101 individuals met eligibility criteria and were randomized to the RCT (see Figure 1, Tables 2 and 3). The sample was predominantly female (75%), most had attended tertiary education and 59% were currently employed. Over 50% reported current physical comorbidity, and a similar proportion had a history of psychiatric treatment. Nearly 9 out of 10 reported previously taking >=1 sleep medication.

**Table 2.** Baseline characteristics of participants assigned to digital Cognitive Behavioral Therapy for Insomnia (dCBT-I) or face-to-face Cognitive Behavioral Therapy for Insomnia (FtF CBT-I)

	dCBT-I (*n* = 49)		FtF CBT-I (*n* = 52)	
Age in years, mean (SD)	41.4	(10.5)	41.3	(12.5)
Sex, *n* (%)				
Female	35	(71%)	41	(79%)
Male	14	(29%)	11	(21%)
Marital status, *n* (%)				
Married or cohabiting	30	(61%)	31	(60%)
Never married, divorced or separated	19	(39%)	21	(40%)
Education attainment, *n* (%)				
Below high school	1	(2%)	4	(8%)
Completed high school	16	(33%)	16	(31%)
College or higher	32	(65%)	32	(62%)
Employment status, *n* (%)				
Full or part time employment	31	(63%)	29	(56%)
Unemployed seeking work	0	(0%)	1	(2%)
Sick leave or disability pension	14	(29%)	15	(29%)
Student	4	(8%)	7	(13%)
Duration of insomnia, years (SD)	12.6	(11.5)	13.0	(11.4)
Comorbidities				
>=1 Physical disorder, *n* (%)	25	(51%)	30	(58%)
Current psychiatric outpatient, *n* (%)	10	(20%)	10	(19%)
Previous psychiatric treatment, *n* (%)	26	(53%)	25	(48%)
Prescribed sleep medications				
Current sleep medication, *n* (%)	31	(63%)	33	(64%)
Previous sleep medication, *n* (%)	42	(86%)	46	(89%)
Number of previous sleep medications, mean (SD)	2.5	(1.7)	2.5	(1.6)

**Table 3.** Self-reported mental disorders at baseline as identified by the Psychiatric Disorders Screening Questionnaire of participants allocated to digital Cognitive Behavioral Therapy for Insomnia (dCBT-I) or face-to-face Cognitive Behavioral Therapy for Insomnia (FtF CBT-I)

	dCBT-I (*n* = 49)	FtF CBT-I (*n* = 52)
Comorbid mental disorders, *n* (%)		
Any mental disorder	40 (82%)	40 (77%)
>=1 Affective disorder(s)	16 (33%)	15 (29%)
>=1 Anxiety disorder(s)	39 (80%)	37 (71%)
>=1 Alcohol and/or substance misuse disorder(s)	12 (24%)	9 (17%)
Other mental disorder(s)	7 (14%)	5 (10%)

Fifty-one (of 52) patients assigned to FtF CBT-I completed the course of therapy (98%). One patient was excluded after two sessions of FtF CBT-I by the therapist (when the patient disclosed meeting exclusion criteria, namely working night shifts, and having a severe substance misuse problem). The average number of FtF sessions was 6 (range 3–8 sessions). In the dCBT-I group, 43 of 49 patients (88%) completed 4 or more CBT-I cores, and 31 patients (63%) completed all the cores.

### Non-inferiority analyses on the primary outcome

**Figure 2.**
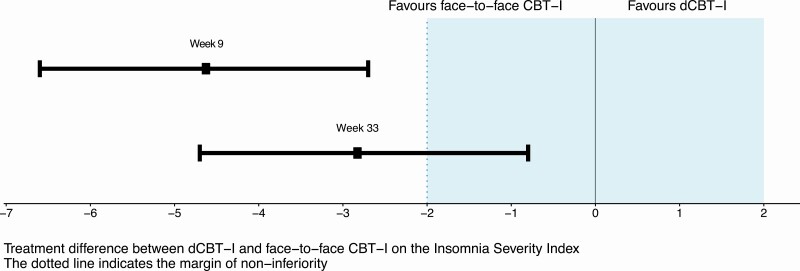
Difference in estimated mean scores on the Insomnia Severity Index between the two interventions and their 95% CI relative to the non-inferiority margin. Figure adapted from the CONSORT statement for non-inferiority trials [17].

There was a significant time effect for both interventions on the ISI score (*p* < 0.001). At the primary endpoint at week 33, participants receiving FtF CBT-I scored 2.8-points lower on the ISI compared with participants receiving dCBT-I (95% CI −4.8 to −0.8; *p* = 0.007; Cohen’s *d =* 0.7). At week 9, the FtF group had a mean ISI score that was 4.6 points lower than the dCBT-I group (95% CI −6.6 to −2.7; *p* < 0.001), Cohen’s *d* = 1.2. As shown in Figure 2, the 95% CI of the estimated mean difference between the two intervention groups at week 33 demonstrates that FtF meet criteria for superiority over dCBT-I (and at 9-week follow-up).

### Planned superiority analyses of secondary outcomes

1. Response and Remission

The response rate at week 9 was significantly higher for FtF CBT-I (*n* = 35/50; 70%) compared with dCBT-I (*n* = 19/44; 43%), which represents a 27% difference in the proportion of responders (95% CI: 7% to 44%; *p* = 0.009). At week 33, there was a marginal change in these response rates (FtF: *n* = 31/48, 65%); dCBT-I: *n* = 19/41, 46%), but the disparity was no longer statistically significant (difference = 18%; 95% CI: −2% to 37%; *p* = 0.084).

The remission rate at week 9 for was significantly higher for FtF CBT-I (*n* = 26/50; 52%) than dCBT-I (*n* = 8/44; 18%), with a 34% difference in the proportion of remitters (95% CI: 15% to 50%; *p* = 0.001). At week 33, the difference remained statistically significant (FtF = 27/48 (56%); dCBT-I = 10/42 (24%)), with a difference of 32%; 95% CI: 12% to 49%; *p* = 0.002.

1. Other Clinical Outcomes

Table 4 summarizes findings for psychological distress, fatigue, dysfunctional beliefs, and self-reported sleep-wake parameters according to group and timing of follow-up. There was a significant time-effect for all secondary outcomes (*p* < 0.001), but no between-group differences. Changes show medium to large within-group ES.

**Table 4.** Primary and secondary outcomes for participants assigned to digital Cognitive Behavioral Therapy for Insomnia (dCBT-I) or face-to-face Cognitive Behavioral Therapy for Insomnia (FtF CBT-I)

	dCBT-I (*n* = 49)				FtF CBT-I (*n* =52)				Difference (group × time)			
	*n*	Mean	SD	*d*	*n*	Mean	SD	*d*	Estimate	95% CI	d*	*P*
ISI												
Baseline	49	19.4	4.1		52	18.4	3.7					
Week 9	44	13.7	7.0	1.4	50	8.4	5.1	2.7	-4.6	−6.6 to −2.7	−1.2	<0.0001
Week 33	41	12.3	6.9	1.7	48	8.9	6.0	2.6	−2.8	−4.8 to −0.8	−0.7	0.007
HADS												
Baseline	49	15.2	6.8		52	12.9	6.6					
Week 9	44	13.6	7.8	0.2	48	9.8	6.6	0.5	−1.9	−3.8 to 0.04	−0.3	0.06
Week 33	41	12.2	8.4	0.4	47	9.0	7.1	0.6	−1.2	−3.2 to 0.7	−0.2	0.2
CFS												
Baseline	49	36.1	6.7		52	35.2	5.8					
Week 9	44	31.6	8.2	0.7	49	29.9	6.4	0.9	−0.9	−3.2 to 1.4	−0.1	0.5
Week 33	41	30.6	8.7	0.8	48	28.3	6.8	1.2	−1.3	−3.7 to 1.0	−0.2	0.3
DBAS-16												
Baseline	48	5.50	1.7		51	5.51	1.9					
Week 9	38	4.00	2.1	0.8	47	3.91	1.9	0.8	−0.3	−0.85 to 0.28	−0.1	0.3
Week 33	31	4.00	2.5	0.8	41	3.42	1.9	1.1	−0.7	−1.3 to −0.1	−0.3	0.02
Sleep diaries												
SOL (min)												
Baseline	49	58.0	48.8		51	51.0	41.7					
Week 9	39	30.0	26.0	0.6	48	24.4	21.6	0.6	0.1	−12.3 to 12.6	<0.1	0.9
Week 33	30	28.6	31.2	0.6	40	27.1	21.9	0.6	4.7	−9.1 to 18.5	0.1	0.5
WASO (min)												
Baseline	49	63.9	46.3		51	53.9	38.1					
Week 9	39	32.2	31.2	0.7	48	27.0	28.1	0.7	−1.9	−17.1 to 12.3	<−0.1	0.8
Week 33	30	34.2	27.8	0.6	40	39.1	53.6	0.4	8.3	−8.5 to 25.2	0.2	0.3
EMA (min)												
Baseline	49	63.2	66.9		51	44.1	33.5					
Week 9	39	21.7	22.7	0.6	48	28.1	30.8	0.5	11.1	−4.7 to 26.9	0.2	0.2
Week 33	30	21.1	18.3	0.6	40	24.3	21.8	0.6	4.8	−12.9 to 22.4	<0.1	0.6
TST (hours)												
Baseline	49	5.23	1.50		51	5.50	1.13					
Week 9	39	5.56	1.59	0.2	48	5.85	1.20	0.3	0.04	−0.40 to 0.47	<0.1	0.9
Week 33	30	6.05	1.45	0.5	40	6.08	1.34	0.5	−0.03	−0.51 to 0.44	<−0.1	0.9
No. awak.												
Baseline	49	2.42	1.58		51	2.56	1.83					
Week 9	39	1.90	2.09	0.3	48	1.52	1.07	0.6	−0.47	−0.95 to 0.01	−0.3	0.06
Week 33	30	1.81	1.59	0.4	40	1.85	1.39	0.4	−0.16	−0.69 to 0.37	<−0.1	0.6
SE (%)												
Baseline	49	63.8	17.1		51	69.0	12.5					
Week 9	39	77.7	16.1	0.8	48	81.4	12.4	1.0	0.03	−5.06 to 5.13	<0.1	0.9
Week 33	30	79.9	14.3	0.9	40	80.3	15.6	0.9	−1.79	−7.42 to 3.83	−0.1	0.5
Sleep med												
Baseline	48	3.5	4.20		51	3.9	4.34					
Week 9	38	2.8	4.18	0.2	47	1.7	3.36	0.5	−1.3	−2.57 to 0.03	0.3	0.06
Week 33	31	3.7	4.53	<0.1	41	2.6	3.80	0.3	−1.5	−2.95 to −0.08	0.4	0.04

Means and SD are descriptive statistics. The difference estimates are results from the baseline-adjusted linear mixed models (positive values favors dCBT-I).

*d*, within-group effect size, Cohen’s *d*; d*, between-group effect size, Cohen’s *d*; ISI, Insomnia Severity Index; HADS, Hospital Anxiety and Depression Scale; CFS, Chalder Fatigue Scale; DBAS, Dysfunctional Beliefs About Sleep Scale—16 items; SOL, sleep onset latency; WASO, wake after sleep onset; EMA, early morning awakening; TST, total sleep time; No. awak., number of nocturnal awakenings; SE, sleep efficiency; Sleep med, number of nights with sleep medication.

### PP analyses

As shown in Supplementary Table S1, the findings of the PP mixed model analyses were substantially the same as the ITT analyses.

## Discussion

This RCT addressed a critical issue about the effectiveness of dCBT-I, namely the lack of data comparing fully automated dCBT-I with FtF CBT-I in a clinical population of patients with insomnia disorder [9]. We hypothesized that dCBT-I would be non-inferior to FtF CBT-I in reducing insomnia severity based on RCT findings indicating that dCBT-I has similar effect sizes to FtF CBT-I [6–8, 16, 34]. However, this RCT did not support this as we found that FtF CBT-I was superior to dCBT-I in reducing insomnia severity in patients referred to a sleep clinic provided by secondary care public mental health services in Norway. This finding is contrary to those reported from studies of other fields, where digital and FtF delivery of CBT has been shown to have similar effectiveness [10–12]. This discrepancy may be due to differences in various elements of the methodology, most notably sampling frames (e.g. recruitment of convenience vs. clinical populations) and the mode of the digital model studied (e.g. therapist guided vs. fully automated). Compared with the three previous trials in insomnia, our findings are similar to a trial of guided dCBT-I compared with FtF CBT-I undertaken in a sample of self-referrals recruited via social and other media [14]. Our findings also add to those reported in a trial in active military personnel, where there was a trend favoring individual FtF CBT-I over fully automated dCBT-I. That trial did not show any significant differences between the two modalities of delivery, but this may be due to low statistical power [15]. However, a third RCT reported that guided dCBT-I was non-inferior to FtF CBT-I delivered in a group format [13]. This is interesting as previous meta-analyses have shown that, whilst group CBT-I is effective [35], it is less effective than individual CBT-I [36]. If confirmed by further trials, this would be an important insight into how dCBT-I might be incorporated into clinical management or stepped-care models. Namely, while dCBT-I is less efficacious than individual FtF CBT-I, it may be as efficacious as group CBT-I and so could offer a viable alternative option, especially as resource availability and cost-benefit considerations would be likely to favor dCBT-I.

Obviously, individuals referred to a sleep clinic for treatment usually have an expectation that they will be offered FtF therapy. So, it is useful to review the data available from this RCT to shed light on the acceptability of dCBT-I and to examine levels of adherence with the intervention. From the CONSORT flowchart, we note that about 9% of the individuals invited to join the RCT declined to participate because they were only willing to accept FtF CBT-I. Although adherence rates for dCBT-I in this RCT are somewhat higher than those reported in previous trials [7, 16, 37], dCBT-I had a lower uptake and completion rate compared with FtF delivered therapy. In the dCBT-I group, 63% completed all elements of the program (six cores) compared with the FtF CBT-I group, where 98% attended all the sessions that were offered. However, our findings regarding difference in insomnia severity according to the group in the ITT analyses were also mirrored by the PP analyses. We suggest that this indicates that the differences we found were not only due to differences in adherence.

Importantly, although FtF CBT-I was superior on the primary outcome of insomnia severity, participants in the two groups did not significantly differ on any of the sleep-wake variables as recorded in the self-report diaries, apart from fewer nights use of sleep medication in FtF CBT-I. In addition, no differences were found in the other secondary outcomes of psychological distress and fatigue. The lack of differences across the secondary variables is particularly intriguing. First, insomnia is not necessarily a condition of having too little sleep, but rather having SOL or WASO which causes daytime distress [2]. These are subtly different concepts and the ISI specifically taps into this by asking individuals to report how severe their problems are (e.g. with SOL and WASO), not the duration of SOL or WASO. Thus, it is possible to improve the insomnia severity beyond improving the sleep-wake variables.

Determining who might be a candidate for fully automated dCBT-I and who might need additional support may help improve its effectiveness and clinical utility [6]. We recently completed a large-scale trial of dCBT-I compared with a control intervention of Patient Education in a self-referred community-based sample in Norway [7]. In that trial, we utilized the same dCBT-I intervention, and the same outcome measures (i.e. ISI, HADS, CFS, and sleep diaries), at the same time points, although the current trial also adds data at week 33. This allows some comparison of the two trials in these two populations. First, we note that about one-third of the participants in the current trial were either on sick leave or had permanent disability pension, compared with about 10% of the community-based sample, indicating that the current sample had lower general levels of functioning. At baseline, the clinical sample also had notably higher levels of daytime fatigue, and higher levels of comorbidities and sleep medication use, while the levels of insomnia severity were similar. This indicates that it may not be the insomnia severity itself, but the complexity of the clinical presentation which could inform clinicians about whether the patient may be a candidate for fully automated dCBT-I or FtF CBT-I, or who might benefit from more personally tailored information. Future research should explore if targeted human support can increase success rates of fully automated dCBT-I in more complex cases. Developments have been made with adaptive strategies to prevent failure in guided digital CBT-I [38]. These may also be integrated into fully automated versions of dCBT-I where “red flags” at various points in the course of dCBT-I may identify individuals who may benefit additional techniques and strategies from the system or even some level of human support.

Although we found that the reduction in insomnia severity in the dCBT-I group was lower in this clinical sample (within-group Cohen’s *d* = 1.4) compared with the above-mentioned community sample (*d* = 2.3) [7], it is noteworthy that the ES for the impact of dCBT-I on insomnia severity was large in the current sample, as was the within-group ES for fatigue and sleep diary variables (Cohen’s *d* > 0.8) whereas the ES on psychological distress was lower (Cohen’s *d* = 0.4). These within-group ES are similar to other trials of dCBT-I [8]. Given these observations, we would argue that the importance of the ES reported for dCBT-I in this RCT should not be underestimated as it exceeds that reported for many other medications or therapies [39] and indicates that this fully automated dCBT-I intervention has important benefits in a clinical sample as well. Thus, future studies should aim to further investigate the effectiveness of dCBT-I in other clinical samples.

The content of CBT-I was similar in the two modalities and participants were exposed to the same major treatment components. Our findings also show that patients in both groups had large reductions in dysfunctional beliefs about sleep during the intervention, with similar effect sizes as typically found in trials of CBT-I [40] and no difference between the two groups at week 9, indicating that both interventions addressed core therapeutic components of CBT-I. However, at week 33, the FtF intervention was superior to dCBT-I in reducing dysfunctional beliefs. Despite the similarities in therapy components, there were differences in how the therapeutic content was delivered. This is important, as it ensured that each intervention is representative of the content and process of therapy delivery in real-world settings. For instance, FtF is delivered by therapists who of course are allowed to personalize the CBT-I to meet the needs of an individual. This might involve changing the sequence of delivery or the emphasis placed on some interventions. Also, patients and therapists can collaboratively review progress toward the patients’ goals (regarding overcoming their sleep problems) and more options to tailor the CBT-I to the individual and/or greater flexibility in the number of sessions offered and/or overall duration of therapy. Obviously, whilst patients may benefit from the convenience of engaging with dCBT-I (flexibility in scheduling self-appointments, etc.), the process is fully automated and patients do not have the option of more subtle or individualized delivery of the intervention. Related to this, if the patient has a goal to taper sleep medication use, therapists can offer an individualized plan whereas dCBT-I does not have a specific module for this. Although it has previously been shown that use of sleep medication is reduced after dCBT-I [7], it is possible that the magnitude of the decrease is greater with FtF CBT-I; which would explain (at least in part) the difference in the use of sleep medication between FtF and dCBT-I. Similarly, the sleep restriction intervention followed the same protocol in both modalities, but it was introduced slightly earlier in FtF CBT-I (in session 1) compared with “Core 2” in dCBT-I. Whilst it is unlikely that this difference affected outcomes during the follow-up phase (at week 33), it may explain larger immediate treatment effects in the FtF group (at week 9). Overall, these subtle differences in the delivery of each therapy program may have contributed to the reported differences between dCBT-I and FtF CBT-I in reducing insomnia severity despite no differences in sleep-wake variables from the sleep diaries. Moreover, the option to personalize CBT-I more when using FtF therapy and the ability of therapists to adapt the interventions to suit the needs of each case in a flexible manner may be a contributing factor that helps to explain why more individuals completed the FtF condition.

Strengths of the current trial include the use of a non-inferiority design with stringent criteria alongside a more rigorous methodology than some earlier publications on dCBT-I (e.g. we used a clinical screening interview, targeted clinical cases of DSM-5 insomnia disorder, and employed of objective tests to assess sleep apnea, such as oximetry), the recruitment of a clinically representative sample of patients who reported a range of comorbidities that are typically associated with insomnia, use of established clinical professionals with long-standing experience in FtF CBT-I as the trial therapists and low levels of missing data. However, we acknowledge several limitations to this RCT.

For example, all the patients participated in an initial diagnostic interview that was undertaken by the CBT-I therapists. Although there was no possibility that the therapists could influence the randomization procedure, it can be viewed as a weakness in the trial design (e.g. post-interview allocation to dCBT-I could seem less acceptable to a participant because they had prior contact with the FtF therapists). Importantly, the selection of a 2-point non-inferiority margin was different from the 4-point non-inferiority margin on the ISI score used in other non-inferiority trials of CBT-I [13, 30], and the 1.67-point margin in ISI change used in a recent non-inferiority trial comparing different modalities of CBT-I delivery [41]. Thus, there is currently no agreed-upon margin of non-inferiority in the literature. We based our argument for this 2-point difference on an a priori assumption about what we would consider a clinically relevant difference (i.e. Cohen’s *d* = 0.5). This difference is also congruent with a meta-analysis by Norman et al. who concluded that *d* = 0.5 seems to be a universal threshold for determining the minimally important difference for health-related quality of life [42]. However, neither a 4-point non-inferiority margin nor a 1.67-point margin, would not have changed the conclusions from the current trial as the 95% CI of the estimated mean difference on the ISI was −4.8 to −0.8 at follow-up. Because the 95% CI includes the margin of non-inferiority we cannot conclude about non-inferiority. Still, the 95% CI is wholly outside 0, we can conclude that FtF CBT-I is superior to dCBT-I. We did not find differences for any of the secondary outcomes, apart from sleep medication use and long-term dysfunctional beliefs. This could be because the trial was not powered to detect small differences in the secondary outcomes. The lack of a third arm with a control group prohibits strong conclusions about the effects of each of the interventions, but there was a significant reduction in scores on all measures from baseline to follow-up for both interventions, and previous research has demonstrated that both FtF CBT-I and dCBT-I is effective compared to control conditions [34]. The description of comorbid mental disorders was based on a self-report of symptoms using the PDSQ. The assessment does not consider any accompanying functional impairment nor does it include any clinical evaluations of social factors, the patient history, and current presentation. As such, the PDSQ findings are likely to be an over-estimate of the number of patients with mental disorders, and the conditions reported do not necessarily meet specific diagnostic criteria for the identified problem [23]. Some evidence to support this hypothesis can be derived from data collected regarding substance use disorders which were evaluated using the PDSQ at baseline but also by clinical interview (at the intake assessment). This discrepancy between clinician evaluation and self-reported substance use could be a result of the PDSQ not assessing functional impairment due to substance misuse, but also those patients did not fully disclose their substance use during the interview. Other objective measures (such as blood tests, etc.) were not available to this study, so currently, we cannot determine which explanation is most plausible. Moreover, the baseline self-reported data on the PDSQ was unavailable to the therapists performing the diagnostic assessment. The dCBT-I participants had access to the intervention for 6 months and we do not know the actual time the patients spent on each module in the dCBT-I condition. It is possible that the continued improvement seen in the dCBT-I group between weeks 9 and 33, is caused by the patients continuing to use dCBT-I in this period. We also do not have data on how many patients in the dCBT-I who wanted to discontinue hypnotic medication and went on to discuss this with their primary care physician after being presented with this information during dCBT-I.

## Conclusion

At the primary endpoint at week 33, in a direct comparison of differences between dCBT-I and individual FtF CBT-I in a clinical sample, the result is inconclusive regarding the possible inferiority or non-inferiority of dCBT-I over FtF CBT-I, but dCBT-I performed significantly worse than FtF CBT-I. At week 9, dCBT-I was inferior to FtF CBT-I as the 95% CI was fully outside the non-inferiority margin. However, it is noteworthy that both groups demonstrated a statistically significant and clinically meaningful reduction in insomnia severity. Furthermore, the two modalities of CBT-I did not differ in regard to outcomes related to sleep-wake variables, psychological distress, or fatigue, but dCBT-I performed significantly worse than FtF CBT-I in reducing sleep medication and long-term levels of dysfunctional beliefs. Our findings suggest that most, but not all, patients referred to secondary care clinical services for an insomnia disorder will accept a trial of dCBT-I and the majority of those who commence the intervention will complete the intervention. However, as the benefits of dCBT-I are attenuated compared with FtF CBT-I, we suggest that further research may be useful to shed light on how to optimize the delivery of dCBT-I and the selection of recipients most likely to benefit.

## Supplementary Material

zsab185_suppl_Supplementary_Material

## Data Sharing

De-identified data that underlie the results reported in this article will be available to researchers from accredited research institutions. Access to data will be limited to investigators who provide a methodologically sound proposal and will be limited to a specified time period (commencing about 3 months after publication of this article and ending after 5 years). To ensure compliance with the General Data Protection Regulation, data processing must be covered by the European Commission’s standard contractual clauses for the transfer of personal data, which must be signed by the data requesters. Proposals and requests for data access should be directed to the corresponding author. User-friendly output from the trial will be disseminated to patient advocacy and other relevant organizations.
