# Supplementary material for: Mode of delivery of Cognitive Behavioral Therapy for Insomnia: a randomized controlled non-inferiority trial of digital and face-to-face therapy
Source: Sleep. 2021 Aug 6;44(12):zsab185. doi: 10.1093/sleep/zsab185 (PMC8664599; doi:10.1093/sleep/zsab185)
Supplement: zsab185_suppl_Supplementary_Material [file zsab185_suppl_supplementary_material.docx]

**Title:** *Mode of delivery of* *Cognitive Behaviour Therapy for Insomnia: A randomized controlled non-inferiority trial of digital and face- to-face therapy*

Håvard Kallestad^1,2*^, Jan Scott^2,3^, Øystein Vedaa^2,4^, Stian Lydersen^2^, Daniel Vethe^2^, Gunnar Morken^1,2^, Tore Charles Stiles^5^; Børge Sivertsen^2,4,6^, Knut Langsrud^1,2^.

^1.^ Division of Mental Health Care, St Olavs University Hospital, Trondheim, Norway

^2.^ Department of Mental Health, Norwegian University of Science and Technology, Trondheim, Norway

^3.^ Institute of Neuroscience, Newcastle University, Newcastle, United Kingdom

^4.^ Department of Health Promotion, Norwegian Institute of Public Health, Bergen, Norway

^5.^ Department of Psychology, Norwegian University of Science and Technology, Trondheim, Norway

^6.^ Department of Research and Innovation, Helse-Fonna HF, Haugesund, Norway

*Corresponding author

Håvard Kallestad

Division of Mental Health Care

Department of Research and Development

PO Box 3250 Torgarden

NO-7033 Trondheim, Norway.

Tel: +47 72823030

E-mail: [havard.kallestad@ntnu.no](mailto:havard.kallestad@ntnu.no)

**Table S1. Per protocol (PP) analyses: Primary and secondary outcomes for participants allocated to digital Cognitive Behavioral Therapy for Insomnia (dCBT-I) or face-to-face Cognitive Behavioral Therapy for Insomnia (FtF CBT-I).**

|  | **dCBT-I (n=31)** | | |  | **FtF CBT-I (n=51)** | | |  | **Difference (group x time)** | | | | |
| --- | --- | --- | --- | --- | --- | --- | --- | --- | --- | --- | --- | --- | --- |
|  | *n* | *Mean* | *SD* |  | *n* | *Mean* | *SD* |  | *Estimate* | *95% CI* | *p* | |  |
| **ISI** |  |  |  |  |  |  |  |  |  |  |  |  |  |
| Baseline | 31 | 20.0 | 3.8 |  | 51 | 18.3 | 3.8 |  |  |  |  |  |  |
| Week 9 | 30 | 13.7 | 6.8 |  | 50 | 8.4 | 5.1 |  | -4.3 | -6.4 to -2.2 | <0.0001 |  |  |
| Week 33 | 28 | 13.1 | 6.8 |  | 48 | 8.9 | 6.0 |  | -3.4 | -5.6 to -1.2 | 0.002 |  |  |
|  |  |  |  |  |  |  |  |  |  |  |  |  |  |
| **HADS** |  |  |  |  |  |  |  |  |  |  |  |  |  |
| Baseline | 31 | 14.6 | 7.1 |  | 51 | 13.0 | 6.6 |  |  |  |  |  |  |
| Week 9 | 30 | 13.5 | 8.1 |  | 48 | 9.8 | 6.6 |  | -2.0 | -4.1 to 0.2 | 0.08 |  |  |
| Week 33 | 28 | 11.3 | 8.4 |  | 47 | 9.0 | 7.1 |  | -0.7 | -2.9 to 1.5 | 0.5 |  |  |
|  |  |  |  |  |  |  |  |  |  |  |  |  |  |
| **CFS** |  |  |  |  |  |  |  |  |  |  |  |  |  |
| Baseline | 31 | 36.1 | 7.0 |  | 51 | 35.4 | 5.8 |  |  |  |  |  |  |
| Week 9 | 30 | 30.8 | 7.8 |  | 49 | 29.9 | 6.4 |  | -0.1 | -2.6 to 2.4 | 0.9 |  |  |
| Week 33 | 28 | 29.8 | 8.6 |  | 48 | 28.3 | 6.8 |  | -0.6 | -3.2 to 2.0 | 0.7 |  |  |
|  |  |  |  |  |  |  |  |  |  |  |  |  |  |
| **DBAS-16** |  |  |  |  |  |  |  |  |  |  |  |  |  |
| Baseline | 31 | 5.48 | 1.6 |  | 51 | 5.50 | 1.9 |  |  |  |  |  |  |
| Week 9 | 28 | 3.94 | 2.0 |  | 47 | 3.91 | 1.9 |  | -0.03 | -0.7 to 0.6 | 0.9 |  |  |
| Week 33 | 22 | 4.39 | 2.4 |  | 41 | 3.42 | 1.9 |  | -0.8 | -1.5 to -0.2 | 0.01 |  |  |
|  |  |  |  |  |  |  |  |  |  |  |  |  |  |
| **Sleep diaries** |  |  |  |  |  |  |  |  |  |  |  |  |  |
| SOL (min) |  |  |  |  |  |  |  |  |  |  |  |  |  |
| Baseline | 31 | 65.1 | 56.4 |  | 51 | 51.1 | 42.1 |  |  |  |  |  |  |
| Week 9 | 28 | 28.9 | 23.6 |  | 48 | 24.4 | 21.6 |  | 6.1 | -7.7 to 19.9 | 0.4 |  |  |
| Week 33 | 22 | 32.2 | 27.7 |  | 40 | 27.1 | 21.9 |  | 10.8 | -4.4 to 26.0 | 0.2 |  |  |
|  |  |  |  |  |  |  |  |  |  |  |  |  |  |
| WASO (min) |  |  |  |  |  |  |  |  |  |  |  |  |  |
| Baseline | 31 | 67.6 | 50.4 |  | 51 | 54.2 | 38.5 |  |  |  |  |  |  |
| Week 9 | 28 | 35.1 | 34.9 |  | 48 | 27.0 | 28.1 |  | -3.0 | -20.3 to 14.2 | 0.7 |  |  |
| Week 33 | 22 | 34.1 | 30.6 |  | 40 | 39.1 | 53.6 |  | 8.3 | -10.8 to 27.4 | 0.4 |  |  |
|  |  |  |  |  |  |  |  |  |  |  |  |  |  |
| EMA (min) |  |  |  |  |  |  |  |  |  |  |  |  |  |
| Baseline | 31 | 65.3 | 78.7 |  | 51 | 44.8 | 33.6 |  |  |  |  |  |  |
| Week 9 | 28 | 16.3 | 14.5 |  | 48 | 28.1 | 30.8 |  | 17.3 | -0.5 to 35.1 | 0.06 |  |  |
| Week 33 | 22 | 18.4 | 16.5 |  | 40 | 24.3 | 21.8 |  | 7.0 | -12.8 to 26.8 | 0.5 |  |  |
|  |  |  |  |  |  |  |  |  |  |  |  |  |  |
| TST (hours) |  |  |  |  |  |  |  |  |  |  |  |  |  |
| Baseline | 31 | 5.15 | 1.50 |  | 51 | 5.52 | 1.14 |  |  |  |  |  |  |
| Week 9 | 28 | 5.64 | 1.67 |  | 48 | 5.85 | 1.20 |  | -0.08 | -0.6 to 0.4 | 0.7 |  |  |
| Week 33 | 22 | 6.04 | 1.58 |  | 40 | 6.08 | 1.34 |  | 0.03 | -0.5 to 0.4 | 0.9 |  |  |
|  |  |  |  |  |  |  |  |  |  |  |  |  |  |
| No. awak. |  |  |  |  |  |  |  |  |  |  |  |  |  |
| Baseline | 31 | 2.26 | 1.57 |  | 51 | 2.51 | 1.81 |  |  |  |  |  |  |
| Week 9 | 28 | 1.89 | 2.29 |  | 48 | 1.52 | 1.07 |  | -0.6 | -1.2 to -0.1 | 0.02 |  |  |
| Week 33 | 22 | 1.83 | 1.74 |  | 40 | 1.85 | 1.39 |  | -0.3 | -0.9 to 0.3 | 0.3 |  |  |
|  |  |  |  |  |  |  |  |  |  |  |  |  |  |
| SE (%) |  |  |  |  |  |  |  |  |  |  |  |  |  |
| Baseline | 31 | 62.3 | 19.8 |  | 51 | 68.9 | 12.6 |  |  |  |  |  |  |
| Week 9 | 28 | 78.6 | 16.9 |  | 48 | 81.4 | 12.4 |  | -1.6 | -7.4 to 4.1 | 0.6 |  |  |
| Week 33 | 22 | 80.0 | 15.7 |  | 40 | 80.3 | 15.6 |  | -2.2 | -8.5 to 4.1 | 0.5 |  |  |
|  |  |  |  |  |  |  |  |  |  |  |  |  |  |
| Sleep med |  |  |  |  |  |  |  |  |  |  |  |  |  |
| Baseline | 31 | 3.48 | 4.14 |  | 50 | 3.78 | 4.29 |  |  |  |  |  |  |
| Week 9 | 28 | 3.04 | 4.26 |  | 47 | 1.70 | 3.36 |  | -1.4 | -2.8 to 0.01 | 0.05 |  |  |
| Week 33 | 22 | 3.59 | 4.46 |  | 40 | 2.56 | 3.80 |  | -1.2 | -2.8 to 0.3 | 0.1 |  |  |

Notes:

Means and SD are descriptive statistics. The difference estimates are results from the baseline adjusted linear mixed models (positive values favors dCBT-I).

ISI = Insomnia Severity Index

HADS = Hospital Anxiety and Depression Scale

CFS = Chalder Fatigue Scale

DBAS-16 = Dysfunctional Beliefs and Attitudes about Sleep scale, 16 items

SOL = Sleep Onset Latency

WASO = Wake After Sleep Onset

EMA = Early Morning Awakening

TST = Total Sleep Time

No. awak = Number of nocturnal awakenings

SE = Sleep Efficiency

Sleep med = Number of nights with sleep medication


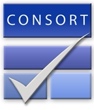
CONSORT 2010 checklist of information to include when reporting a randomised trial*

| Section/Topic | Item No | Checklist item | Reported on page No |
| --- | --- | --- | --- |
| Title and abstract | | | |
|  | 1a | Identification as a randomised trial in the title | 1 |
|  | 1b | Structured summary of trial design, methods, results, and conclusions (for specific guidance see CONSORT for abstracts) | 2 |
| Introduction | | | |
| Background and objectives | 2a | Scientific background and explanation of rationale | 4-6 |
|  | 2b | Specific objectives or hypotheses | 6 |
| Methods | | | |
| Trial design | 3a | Description of trial design (such as parallel, factorial) including allocation ratio | 6 |
|  | 3b | Important changes to methods after trial commencement (such as eligibility criteria), with reasons | n/a |
| Participants | 4a | Eligibility criteria for participants | 6-7 |
|  | 4b | Settings and locations where the data were collected | 6 |
| Interventions | 5 | The interventions for each group with sufficient details to allow replication, including how and when they were actually administered | 8-9 |
| Outcomes | 6a | Completely defined pre-specified primary and secondary outcome measures, including how and when they were assessed | 9-10 |
|  | 6b | Any changes to trial outcomes after the trial commenced, with reasons | n/a |
| Sample size | 7a | How sample size was determined | 10 |
|  | 7b | When applicable, explanation of any interim analyses and stopping guidelines | n/a |
| Randomisation: |  |  |  |
| Sequence generation | 8a | Method used to generate the random allocation sequence | 7-8 |
|  | 8b | Type of randomisation; details of any restriction (such as blocking and block size) | 7-8 |
| Allocation concealment mechanism | 9 | Mechanism used to implement the random allocation sequence (such as sequentially numbered containers), describing any steps taken to conceal the sequence until interventions were assigned | 7-8 |
| Implementation | 10 | Who generated the random allocation sequence, who enrolled participants, and who assigned participants to interventions | 7-8 |
| Blinding | 11a | If done, who was blinded after assignment to interventions (for example, participants, care providers, those assessing outcomes) and how | 10 |
|  | 11b | If relevant, description of the similarity of interventions | n/a |
| Statistical methods | 12a | Statistical methods used to compare groups for primary and secondary outcomes | 10-12 |
|  | 12b | Methods for additional analyses, such as subgroup analyses and adjusted analyses | 10-12 |
| Results | | | |
| Participant flow (a diagram is strongly recommended) | 13a | For each group, the numbers of participants who were randomly assigned, received intended treatment, and were analysed for the primary outcome | 12 + Figure 1 |
|  | 13b | For each group, losses and exclusions after randomisation, together with reasons | Figure 1 |
| Recruitment | 14a | Dates defining the periods of recruitment and follow-up | 6 |
|  | 14b | Why the trial ended or was stopped | n/a |
| Baseline data | 15 | A table showing baseline demographic and clinical characteristics for each group | Table 2 & 3: 29-31 |
| Numbers analysed | 16 | For each group, number of participants (denominator) included in each analysis and whether the analysis was by original assigned groups | Table 4: 32-35 and table S1 |
| Outcomes and estimation | 17a | For each primary and secondary outcome, results for each group, and the estimated effect size and its precision (such as 95% confidence interval) | 12-13, Table 4: 32-35 and table S1 |
|  | 17b | For binary outcomes, presentation of both absolute and relative effect sizes is recommended | n/a |
| Ancillary analyses | 18 | Results of any other analyses performed, including subgroup analyses and adjusted analyses, distinguishing pre-specified from exploratory | 12-13 and table S1 |
| Harms | 19 | All important harms or unintended effects in each group (for specific guidance see CONSORT for harms) | n/a |
| Discussion | | | |
| Limitations | 20 | Trial limitations, addressing sources of potential bias, imprecision, and, if relevant, multiplicity of analyses | 14-20 |
| Generalisability | 21 | Generalisability (external validity, applicability) of the trial findings | 14-20 |
| Interpretation | 22 | Interpretation consistent with results, balancing benefits and harms, and considering other relevant evidence | 14-20 |
| Other information | | |  |
| Registration | 23 | Registration number and name of trial registry | 6 |
| Protocol | 24 | Where the full trial protocol can be accessed, if available | n/a |
| Funding | 25 | Sources of funding and other support (such as supply of drugs), role of funders | 21 |

*We strongly recommend reading this statement in conjunction with the CONSORT 2010 Explanation and Elaboration for important clarifications on all the items. If relevant, we also recommend reading CONSORT extensions for cluster randomised trials, non-inferiority and equivalence trials, non-pharmacological treatments, herbal interventions, and pragmatic trials. Additional extensions are forthcoming: for those and for up to date references relevant to this checklist, see [www.consort-statement.org](http://www.consort-statement.org).
